# The complete plastid genome of *Thrixspermum centipeda* (Orchidaceae, Aeridinae)

**DOI:** 10.1080/23802359.2021.1906171

**Published:** 2021-03-26

**Authors:** Meng-Wei Chi, Ding-Kun Liu, Cheng-Yuan Zhou, Ming-He Li, Si-Ren Lan

**Affiliations:** Key Laboratory of National Forestry and Grassland Administration for Orchid Conservation and Utilization, College of Landscape Architecture, Fujian Agriculture and Forestry University, Fuzhou, China

**Keywords:** Aeridinae, chloroplast genome, phylogeny, *Thrixspermum*

## Abstract

The complete plastid genome of the type species of *Thrixspermum*, *Th. centipeda*, was determined and analyzed in this work. The plastome was 147,888 bp in length with 85,899 bp of the large single-copy (LSC) region, 11,055 bp of the small single-copy (SSC) region and 25,467 bp of the invert repeats (IR) regions. The genome contained 120 genes, including 74 protein-coding genes, 38 tRNA genes, and 8 rRNA genes. Phylogenetic analysis divided 18 Aeridinae plastomes into four groups, and *Th. centipeda* was sister to *Th. tsii*.

The *Thrixspermum* comprises about 160 species and is one of the largest genera of Aeridinae (Chase et al. 2015). This genus is distributed in the subtropical and tropical regions from Asia to Australia (Pridgeon et al. 2014). The intergeneric and intrageneric phylogenetic relationships of *Thrixspermum* remain unresolved (Pridgeon et al. 2014; Zou et al. 2015). Here, the plastid genome of the type species of *Thrixspermum*, *Th. centipeda*, was sequenced and assembled for phylogeny and evolution research.

Fresh leaf sample of *Th. centipeda* was acquired from Menghai County, Yunnan Province of China (21°55′N, 100°07′E). The voucher specimen deposited at Fujian Agriculture and Forestry University (specimen code MH Li or081). DNA extraction, library constructing, sequencing and data filtering were carried out following the methods described by Liu et al. (2019). The paired-end reads were filtered with GetOrganelle pipe-line (Jin et al. 2020) to get plastid-like reads using the reference of *Th. japonicum* (KX871234). The plastid-like reads were assembled by SPAdes version 3.10 (Bankevich et al. 2012) to get the ‘fastg.’ The final ‘fastg’ files were filtered by the script of GetOrganelle to obtain pure plastid contigs, and the De Brujin graphs were viewed and edited by Bandage (Wick et al. 2015). Assembled plastid genome was annotated using the reference of *Th. japonicum* by GENEIOUS v11.1.5 (Biomatters Ltd., Auckland, New Zealand) (Kearse et al. 2012). According to Zou et al. (2015) and Liu et al. (2020), a matrix of 18 representative species of Aeridinae and three outgroup species (*Calanthe triplicata*, *C. davidii* and *Cattleya crispata*) were aligned using MAFFT v7.307 (Katoh and Standley 2013). The phylogenetic tree was constructed by the maximum likelihood software IQ-TREE (Nguyen et al. 2015) based on the complete plastid genomes, and branch supports with the ultrafast bootstrap (Hoang et al. 2018).

The complete plastid genome sequence of *Th. centipeda* (GenBank accession MW057769) was 147,888 bp in length, with a large single-copy (LSC) region of 85,899 bp, a small single-copy (SSC) region of 11,055 bp, and a pair of inverted repeats (IR) regions of 25,467 bp. The complete genome GC content was 36.4% (LSC, 33.5%; SSC, 27.2%; IR, 43.2%) and the plastome contained 120 genes, including 74 protein-coding genes, 38 tRNA genes, and 8 rRNA genes.

The phylogenetic analysis divided *Th. centipeda* and 17 Aeridinae plastomes into four groups, and *Th. centipeda* was sister to *Th. tsii* with full support (Figure 1). Clade I contained four species of *Phalaenopsis*. Clade II comprised three species of *Thrixspermum* and it was sister to clade III plus clade IV. Clade III contained two species of *Gastrochilus* and *Pelatantheria scolopendrifolia*. Clade IV contained two species of *Vanda* and six species of *Holcoglossum*.

**Figure 1.**
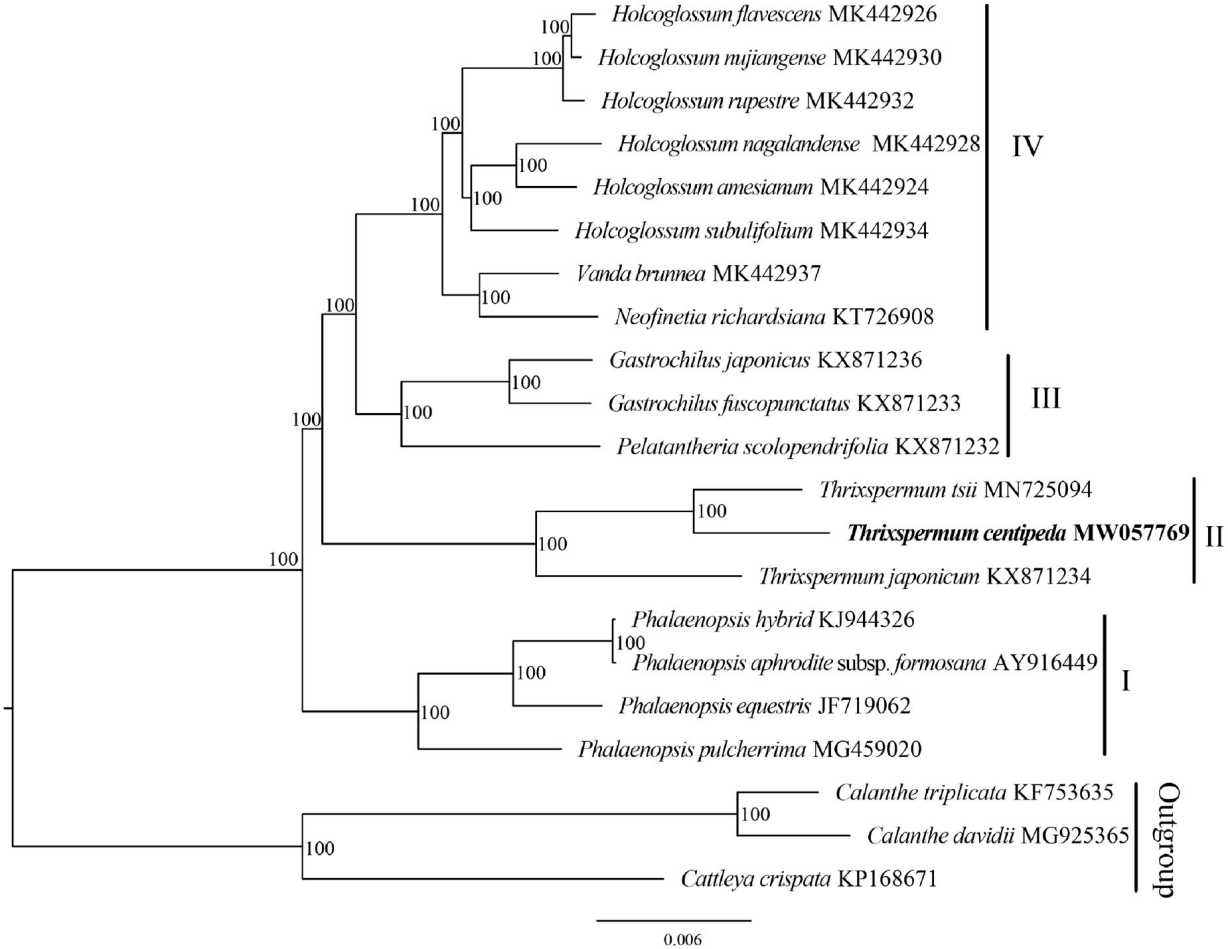
The Maximum-Likelihood (ML) tree based on the plastid genomes of *Th. centipeda* and the other 17 species of Aeridinae. Numbers near the nodes mean bootstrap support value.

## Data Availability

The complete plastid genome of *Th. centipeda* is available in NCBI GenBank database (https://www.ncbi.nlm.nih.gov) with the accession number is MW057769. It is also available at Mendeley Data (http://dx.doi.org/10.17632/wj96wzmsm6.1).
